# Complete Response of Metastatic Melanoma to Local Radiation and Immunotherapy: 6.5 Year Follow-Up

**DOI:** 10.7759/cureus.3723

**Published:** 2018-12-12

**Authors:** Paulina M Gutkin, Susan M Hiniker, Susan M Swetter, Sunil A Reddy, Susan J Knox

**Affiliations:** 1 Radiation Oncology, Stanford University Medical Center, Stanford, USA; 2 Dermatology, Stanford University Medical Center, Stanford, USA; 3 Oncology, Stanford University Medical Center, Stanford, USA

**Keywords:** abscopal effect, ipilimumab, metastatic melanoma, radiation therapy, immunotherapy in cancer

## Abstract

The combined use of immunotherapy and radiation therapy is emerging as a potentially effective treatment for patients with immunogenic tumors such as melanoma; however, evidence for long-term treatment outcomes is lacking. Herein, we summarize our previously described case study of a patient with metastatic melanoma treated with two cycles of ipilimumab, followed by stereotactic body radiotherapy to two of seven liver metastases, with two additional cycles of ipilimumab. In the longest follow-up to date, we report a successful treatment outcome at 6.5 years. Our patient remains in complete remission, with no evidence of disease or recurrence 6.5 years after treatment. He continues to manage chronic hypophysitis developed secondary to immunotherapy and has developed osteopenia from prolonged systemic glucocorticoid use. The use of radiotherapy in combination with targeted immune therapy appears to be an effective treatment strategy, with long-lasting efficacy.

## Introduction

Melanoma is a highly immunogenic cancer, and its ability to induce a systemic immune response has made it ideal for immunotherapy treatment. Increasing numbers of reports in the literature have demonstrated the safety and efficacy of combined radiation treatment (RT) with systemic immunotherapy as a means of inducing a clinically meaningful anti-melanoma immune response with an abscopal response at sites of disease distant from those treated with local RT [1-5].

Herein, we present the 6.5 year follow-up of a patient with metastatic (stage IV) melanoma. This case illustrates a promising successful long-term treatment outcome of combined immunotherapy and high-dose stereotactic body radiotherapy in metastatic melanoma. We discuss current findings regarding the abscopal effect as well as current literature exploring the mechanism of action for the abscopal effect.

## Case presentation

In our initial case report [1], we described the case of a 57-year-old man diagnosed with stage IIA cutaneous melanoma in November 2007 following biopsy of a melanocytic lesion on the left posterior arm that revealed ulcerated primary melanoma with Breslow thickness 1.75 mm and a mitotic index of 1/mm^2^.

He underwent wide excision of the primary lesion followed by completion lymph node dissection (LND) of the left axillary basin following what was initially deemed a positive sentinel lymph node biopsy, although histology from the LND favored a diagnosis of capsular nevi within several regional lymph nodes (LNs) (ie, pathologic stage IIA, T2b melanoma). The patient remained disease-free for three years, when an in-transit metastasis was detected near the primary site. Following excision of the lymphatic metastasis, he was treated with adjuvant radiotherapy of 50 Gy in 20 fractions to the left posterior arm, followed by one month of adjuvant systemic therapy with high-dose interferon (IFN). One year later, he experienced a second local in-transit recurrence, with positron emission tomography–computed tomography (PET-CT) imaging and tissue confirmation of two to three metastatic melanoma lesions in the liver. BRAF testing on the hepatic metastasis was negative for the V600 mutation.

**Figure 1:**
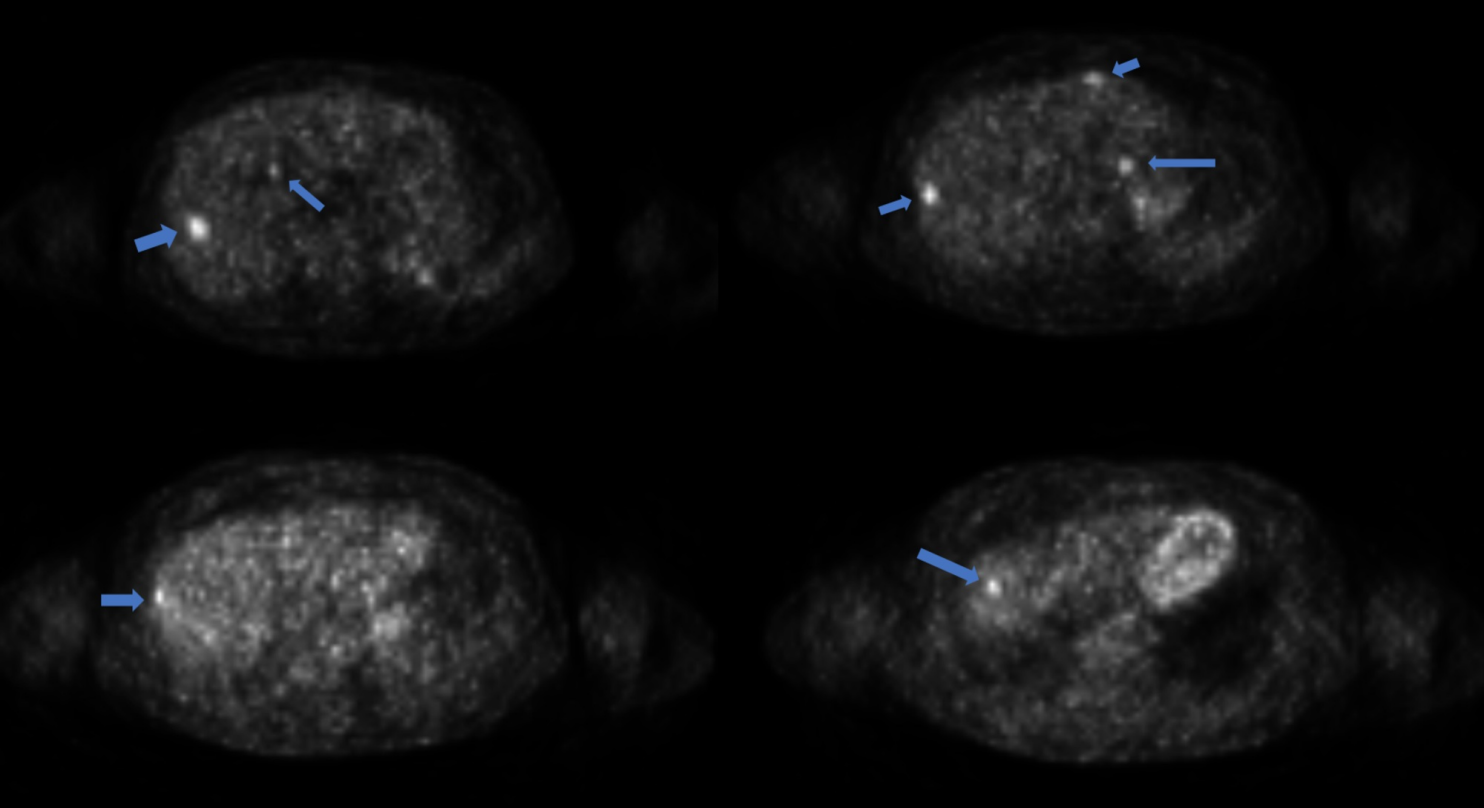
PET-CT scan of liver prior to radiation therapy PET-CT - positron emission tomography-computed tomography

**Figure 2:**
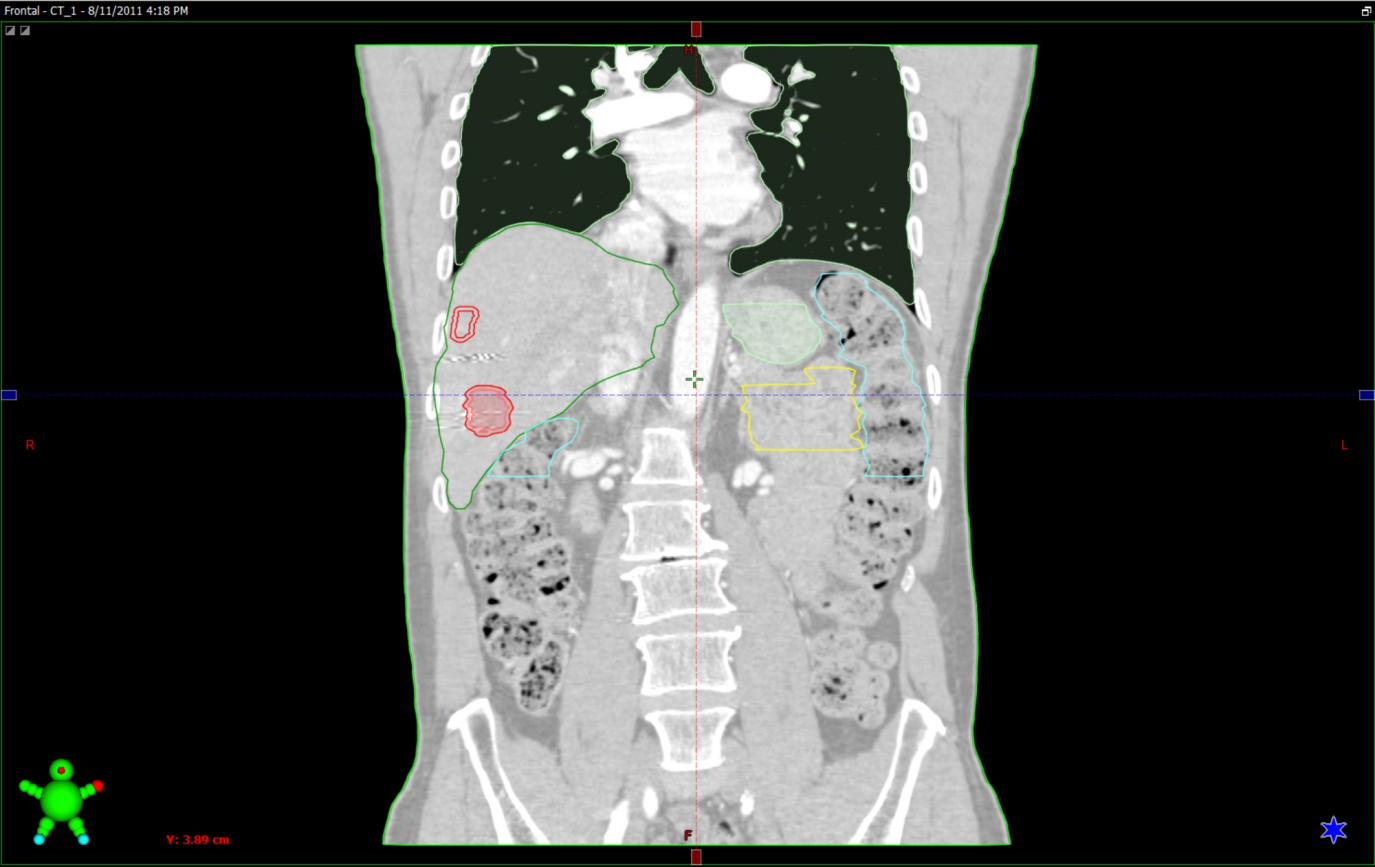
SBRT radiation plan Margins for the two treated liver metastases are outlined in red SBRT - stereotactic body radiotherapy

To induce an anti-tumor immune response that could mediate systemic tumor regression (abscopal effect), planned treatment for the patient included four doses of ipilimumab (anti-CTLA-4) at 3 mg per kilogram of body weight every three weeks, with radiation to begin after two doses. After two cycles of ipilimumab alone, a PET-CT scan showed progression of liver metastases with enlargement of the two previous liver lesions and development of five new hypermetabolic foci in the liver, the largest measuring 2.3 x 2.5 cm (Figure 1). He then was treated with stereotactic body radiotherapy (SBRT) to two of the liver metastases with a total dose of 54 Gy in three fractions (Figure 2). During cycle 3 of ipilimumab, he developed another in-transit melanoma recurrence in his left upper arm, which later completely resolved following initial enlargement consistent with “pseudoprogression.” He also developed hypophysitis that was treated with prednisone. Our previous report concluded at one-year follow-up, in August 2012, with complete resolution of the hypermetabolic lesion in the left arm and all seven hypermetabolic lesions in the liver and no evidence of local or distant disease progression.

Follow-up

**Figure 3:**
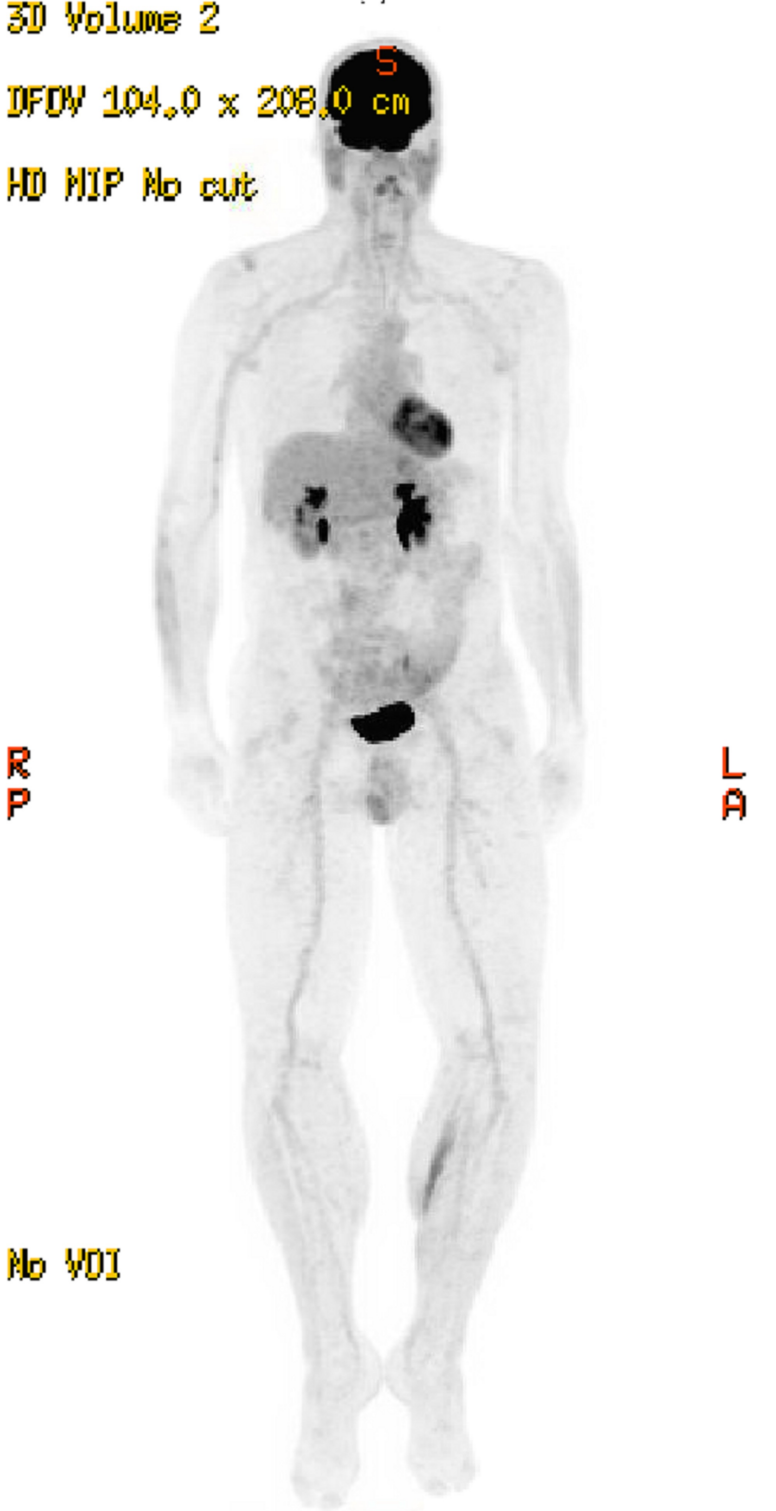
Five-year follow-up MIP scan MIP - maximum intensity projection

Six and a half years after completing treatment, the patient remains disease-free. He continues to be followed every six months, and as of June 2018, he has no clinical or radiologic evidence of disease recurrence. His most recent PET-CT and maximum intensity projection (MIP) scans were performed in 2016 and showed no evidence of disease (Figure 3). His dual-energy X-ray absorptiometry (DEXA) scan T-score was -2.0, which is consistent with osteopenia, a secondary effect of prolonged prednisone use for chronic hypophysitis that he developed from ipilimumab. Symptoms of hypophysitis recurred when he attempted to wean off the prednisone. He therefore remains on a regimen of 3 mg per day. Bisphoshosphonate therapy was recommended, which the patient declined, though he continues to take calcium and vitamin D supplements to manage the osteopenia.

## Discussion

Our patient represents the longest reported follow-up of an abscopal complete response (CR) in metastatic melanoma, remaining without evidence of disease or recurrence for 6.5 years after a combined modality treatment of ipilimumab and radiotherapy to two of his seven metastatic melanoma liver lesions. Reports of this phenomenon in patients with metastatic cancer have increased since Postow et al. first reported it in 2012 [2-6].

Radiation has long been used as a local therapy for cancer and growing evidence shows that irradiation can cause or contribute to regression of distant tumors at non-irradiated sites. The “abscopal effect” is mediated by the induction of a clinically meaningful anti-tumor immune response. There are multiple factors involved in this process. For example, radiation induces upregulation of the major histocompatibility complex (MHC) I surface expression which has been shown to enhance immune responses [7]. MHC I is expressed on all nucleated cells to present intracellular antigenic peptides and is recognized by CD8+ cytotoxic T lymphocytes (CTLs) to signal cell death upon antigen binding. Many tumor cells downregulate MHC I surface expression to evade this major component of the adaptive immune system. A recent study showed that progressive killing of early stage MHC I positive melanoma cells by the CD8+ T-cells selects for melanoma MHC I negative cells through a point mutation resulting in loss of beta2-microglobulin (b2m) [8]. Additionally, local high dose radiation increases production of type I interferon cytokine family, which enhances migration and function of CD8+ T-cells [9]. Upregulation of MHC I expression with radiation in a dose-response manner helps to prevent tumor cell evasion of immune checkpoints and, combined with increased tumor antigen production and enhanced T-cell function, increases susceptibility for targeting by CTLs. Other mechanisms of the immunological effects of radiation have been studied, indicating that radiation enhances high mobility group protein B1 (HMGB1) and FAS surface expression, activates dendritic cells to enhance cross-presentation of tumor antigens, and increases the density of tumor-infiltration lymphocytes [7].

The rarity of cases describing the abscopal effect with RT alone suggest that radiation is not sufficient to induce efficacious systemic antitumor effects in most cases. While radiation promotes immune stimulatory signals, it also has immunosuppressive effects (e.g. stimulating tumor growth factor-b (TGFb) production and upregulating T-regulatory (T-reg) cells) [10]. Therefore, additional immunomodulatory effects are needed to promote effective anti-tumor immune responses. It is well established that co-stimulation is necessary for complete T-cell activation and downstream production of interleukin-2 (IL-2) [11]. CD28 is constitutively expressed on the T-cell surface and binds to the B7 family on the antigen-presenting cell (APC) in conjunction with T-cell receptor (TCR) engagement of the antigen-bound MHC I on the APC. Cytotoxic T-lymphocyte associated protein 4 (CTLA-4) is an inhibitory receptor that is constitutively expressed on regulatory T-cells (Tregs). When upregulated, CTLA-4 binds B7 with higher avidity than CD28, inhibiting co-stimulation of T-cell activation, cell proliferation, and cytokine production [12]. Extensive evidence shows that anti-CTLA-4 overcomes CTLA-4 induced inhibition to enable T-cell activation and can induce an antitumor T-cell response in patients with advanced melanoma [13]. The combination of ipilimumab, a monocolonal IgG1k antibody against CTLA-4, and radiotherapy can result in a synergistic effect, resulting in improved survival in a subset of patients with immunogenic tumors [13].

While the role of ipilimumab in aiding an anti-tumor immune response is promising, it induces immune-related adverse events (irAEs) in up to 64% of patients [14]. Checkpoint blockade with anti-programmed death (PD)-1 monotherapy is associated with fewer irAEs and higher efficacy and has largely supplanted therapy with ipilimumab monotherapy. Autoimmune reactions to a variety of normal tissues have been reported and include dermatitis, colitis, vitiligo, hepatitis, pancreatitis, and endocrinopathies, which can range from mild to severe [15]. Onset of hypophysitis in our patient occurred three months after starting ipilimumab and is manageable with ongoing glucocorticoid replacement. Ipilimumab-induced hypophysitis has been reported in 12%-13% of cases and appears more frequently in men even after adjusting for male predominance in melanoma [16]. A mouse model showed mounting of pituitary autoantibodies following induction of anti-CTLA-4, with formation of complement factors C3, C3d, and C4d involved in the classical immune pathway [17]. Additionally, recent autopsy series showed significantly higher destruction of the anterior pituitary gland in a patient with the highest levels of CTLA-4 antibody than in those who were treated with anti-CTLA-4 but had lower pituitary levels [18]. These reports suggest that, upon CTLA-4 antibody binding to pituitary cells, a combination of inflammatory and autoimmune mechanisms cause activation of the complement system, which contributes to an immune response resulting in hypersensitivity and pituitary cytotoxicity.

The longevity of the remission of this patient demonstrates the potential benefit of combining checkpoint blockade and radiotherapy. Of note, this patient was treated before anti-PD-1 therapy was widely used in melanoma, and he did not receive any additional cancer-directed treatment after completing the four cycles of ipilimumab. Use of a high dose of radiotherapy (54 Gy in three fractions) may have enhanced systemic immune response contributing to effective tumor resolution [19]. The results of our case study support previous clinical observations [2-5]. While these studies have relatively short follow-up (median 22-55 weeks [4,5]), they, in combination with our data and that of others suggest that this combined treatment is a promising therapeutic approach for patients with metastatic melanoma.

In order to optimize this therapy, further investigation into the mechanism of action is needed to understand how best to combine these therapies. Several prospective trials have shown the promise of combining checkpoint blockade with RT to induce a synergistic effect in the treatment of immunogenic cancers [20]; however, more randomized trials are needed to determine which radiotherapy regimens (dose/fraction), sites, and field sizes are most effective. Studies should also address optimization of sequence and include correlative studies for the purposes of identifying biomarkers for use for patient selection and early identification of the generation of an anti-tumor immune response. The study of other immunotherapeutic agents, such as anti-PD-1 and anti-PD-ligand (L1) in combination with RT is an active area of clinical investigation.

## Conclusions

We report long term successful outcomes 6.5 years following combined radiotherapy and immunotherapy in a patient with metastatic melanoma. This patient has had a durable, complete abscopal response, demonstrating the potential of this combined therapy as a noninvasive *in vivo* tumor vaccine strategy.
